# On Digital Percussion and the Cardiac Sign in Carcinoma

**Published:** 1914-03

**Authors:** W. Gordon

**Affiliations:** Hon. Physician to the Royal Devon and Exeter Hospital


### ON DIGITAL PERCUSSION AND THE CARDIAC SIGN IN CARCINOMA.

BY

W. GORDON, M.A., M.D.Cantab., F.R.C.P.,

*Hon. Physician to the Royal Devon and Exeter Hospital.*

---

### THE CARDIAC SIGN.

### *A Summary.*

THE cardiac sign in carcinoma is a remarkable diminution of the area of cardiac dulness in the recumbent posture as determined by digital percussion. In that posture the dulness, in the normal adult, begins above about the third costal cartilage, reaches rightwards as nearly as possible to the mid-sternal line, and measures across about 3 to 3½ inches at the level of the fifth costal cartilage. On the other hand, in the carcinoma patient who presents the sign the cardiac dulness in recumbency begins above about the fourth or fifth costal cartilage, has its rightward edge about half an inch or 1 inch to the left of the mid-sternal line, and measures across less than 2 inches at the level of the fifth costal cartilage. Often it measures less than 1 inch across. Sometimes there is no cardiac dulness at all.

The sign is often associated—by no means always—with a remarkably soft and toneless pulse and feeble heart sounds.

There are three different conditions which may help to explain this diminution in the dulness :—

1. In some cases of carcinoma the heart is small, and this reduction in size has been held to account for the reduction of the dulness. It may sometimes partly account for it, but certainly not invariably, for often the dulness, though narrow in the recumbent posture, is normally or even abnormally broad in the erect.

2. If the loss of elasticity, so common in the skin in cases of carcinoma, affects the lung as well, it is conceivable that ordinary breathing may induce a sort of spurious emphysema, and so lessen the heart-dulness. It is now known that a peculiar form of emphysema not uncommonly occurs in carcinoma—Fenwick found it in 28 per cent. of his cases of gastric carcinoma—and no doubt in many cases this helps to produce the sign.

3. As has just been said, the cardiac sign is often associated with a very soft and toneless pulse and with feeble heart-sounds. If we suppose that the first is due to a deficiency of blood—such as the anæmia so often present would suggest—and that the second shows a flabbiness of the heart muscle comparable to the flabbiness of the skeletal muscles so commonly noticeable in such patients, it is not difficult to imagine a flabby and imperfectly filled heart dropping back, on recumbency, from the anterior chest-wall, more than a normal heart would do. And this is just what seems to occur. For the disparity in area between the heart-dulness in the erect and recumbent positions in carcinoma is usually much greater than that observed either in health or in other diseases. I have found, for instance, a heart-dulness in a case of carcinoma measuring 5 inches across in the erect position narrow to $1\frac{1}{2}$ inches across when the recumbent position was assumed.

Certain limitations exist to the significance of the sign. Thus when any cause is present, tending, like ordinary emphysema, to reduce the area of cardiac dulness, then a very small dulness naturally conveys no special meaning. On the other hand, when there is any well-known cause of enlargement of heart-dulness, such as albuminuria, valvular heart disease, pericarditis, or retraction of lung from phthisis or past pleurisy, then the absence of the sign is equally without significance. Moreover, where the heart is displaced considerably upwards the absence of the sign is unreliable, since the organ has been moved from a wider into a narrower space, in which it is not so free to fall back from the anterior chest-wall on recumbency. And lastly, where a large œsophageal carcinoma has lain directly behind the heart, I have thought that the absence of the cardiac sign was due to the growth pinning the organ forward against the sternum and rib cartilages.

Yet allowing for all these limitations, a very large number of cases remain in which its help is of value. All the cases to which I am now about to allude have been free from such sources of error.

In 1908 I published a series of 103 cases [1], into all of which a suspicion of carcinoma had entered. Of 38 in which a presumable cancer was either naturally accessible to direct examination or was examined at operation or *post-mortem* 89 per cent. showed the sign; of 19 which ended fatally in accordance with a diagnosis of malignancy nearly 90 per cent. showed it. On the other hand, the sign was present in only 24 per cent. of 46 cases which could not be supposed to have been malignant.

Again, last year I dealt with a further series of 73 cases [2] in all of which the diagnosis of carcinoma had to be considered; of 36 which seemed undoubtedly to have been cancerous 83 per cent. gave the sign, whereas of 37 which could not be regarded as having been cancerous only 14 per cent. gave it.

Last month I published a third series of cases [3], containing 42 in which the sign was available, all more or less suspicious as regards cancer; of 18 in which carcinoma was clearly present 88 per cent. showed the sign, whereas of 24 cases which were clearly non-carcinomatous only 8 per cent. gave it.

In all these three series, therefore, a marked contrast has appeared betwixt cancer cases and cases not cancerous, in respect of the frequency of occurrence of the sign. Taking them all together, of a total of 111 cancerous cases 97 (87 per cent.) gave the sign, whereas of a total of 107 non-cancerous cases only 18 (16 per cent.) gave it.

To test further the actual practical utility of the sign, I began on October 1st, 1912, to set down in easily accessible form every diagnosis of "cancer" and "not cancer" which I made in cases which were definitely doubtful when they were submitted to me, and these diagnoses I followed up so far as was possible, in order to discover how many and what sort of mistakes I had been making. At the same time, not more than half a dozen cases were set aside as uncertain. With the help of the cardiac sign, I was only wrong in 6 per cent. in a series of 50, a result which I feel sure I could not have attained without such considerable help [3].

But it has been suggested that the sign is no more than a corroboration of marked wasting. This is untrue, since in non-cancerous wasting, even when extreme, the sign is rarely found, in carcinomatous wasting, even when marked, it is sometimes absent, in cancerous cases which have not wasted at all it is sometimes present, and the diminution of the dulness in cancer cases is not proportional to the wasting [2].

Another suggestion has been that it is partly the result of senility. An examination of the gastric cases in my tables (for clearly in such an inquiry we must limit ourselves to a single organ, as the age incident of carcinoma is not the same in all tissues) lends little support to this view[4].

Thus, whereas of 43 cancerous cases at all ages 37, or about 86 per cent., gave the sign, of 38 of these cases, all at or over 45 years of age, 34, or about 89 per cent., gave it, and of 29 of these, all at or over 55 years of age, 26, or about 89 per cent., gave it; whilst of non-cancerous cases, whereas of 57, at all ages, only 10, or about 17 per cent., gave the sign, of 37 of these cases, all at or over 45 years old, 8, or about 21 per cent., gave it, and of 21 of them, at or over 55, 4, or about 19 per cent., gave it.

I would sum up my experience as follows:—

1. The sign is of practical value.

2. When present in a case of possible carcinoma, the diagnosis of carcinoma should only be rejected after most careful consideration.

3. When present in what must be, if carcinoma at all, a late case of it, its absence strengthens considerably the hope of the absence of carcinoma.

There remains to consider its period of appearance. The importance of an early sign of carcinoma would be great indeed. But here, unhappily, one is not yet in a position to gauge its frequency, because so few cases have presented themselves in an operable stage. Cases are, however, accumulating in which it has appeared in time to enable a successful resection to be carried out. In one such case (cancer of the sigmoid) a well-known surgeon declined to admit that a diagnosis was possible; but it had fortunately been made, which was the reason he had been sent for, and at operation a carcinoma was found and removed; that was six years ago, and there has been no recurrence.

It is possible that the sign may appear earlier in carcinoma of one organ than in carcinoma of another, but as yet the collected cases are too few to safely divide them according to the organs affected, or even to compare its commonness of occurrence in cancers in different situations. Most of my cases have been of carcinoma of the alimentary canal.

In sarcoma I believe that the sign is unreliable, but my observations have been too few to make sure of this.

It is necessary to add that my explanation of the sign has been questioned by Dr. Albert Abrams, of San Francisco, who writes as follows [5] :—

" The cardiac sign of Gordon in cancer has prompted me to study the amplitude of the heart reflex of contraction as influenced by posture.

" Gordon's explanation of the phenomenon is unsatisfactory. It is most probably caused by the elicitation of the heart reflex of contraction by the negative discharge of energy from carcinomata.

" Why is the reflex in question only in evidence in the recumbent posture ? My investigations demonstrated that the heart reflex when evoked has double the amplitude in the recumbent than in the erect posture, owing to the fact that the tone of the cardiac musculature in recumbency is relatively diminished, and in consequence it more readily responds to influences which evoke the reflex."

### Note on Digital Percussion.

It is now ten years since attention was first called to the cardiac sign [6], and seeing how defective are our means of diagnosing internal carcinomata, even late, it might be supposed that it would have been generally used. It certainly has received a wide recognition, but it is equally certainly not in general use. And the inevitable inference is even more important than the sign.

For one of two things must be true. Either the observations above summarised are erroneous, or there must be something amiss with the digital percussion of those who cannot substantiate them.

Naturally I believe my own frequently repeated observations are correct, yet if they were unverifiable by others the presumption would be strong that some fallacy underlay them. They have, however, been verified by others, and the question arises how does digital percussion differ throughout the profession.

I have no sort of doubt why some observers fail to verify the sign. They do not percuss with the requisite precision. This seems a bold statement and certainly needs justifying. A little reflection will, I think, convince most men that there is justification for it. The directions given in every text-book are doubtless sufficiently precise—that the finger percussed should lie flat, evenly and lightly on the chest wall, parallel to the edge of dulness to be defined, that percussion should be from the wrist, and that the percussion finger should be bent, as in piano playing, to deliver its stroke perpendicularly to the chest-wall. But how many percussors follow these directions? To the piano player they are simple, natural and easy, but to others not so trained they require an amount of attention and practice seldom given to them. Consequently, instead of the clear, clearly audible note which digital percussion ought to elicit, whether light or heavy, only a blurred indistinct sound is evoked, quite incapable of exact delimitation of dull or resonant areas. To such percussion the cardiac sign is undiscoverable.

I venture to say, with all deference to those whose fine teaching of medicine is secure against outside petty fault-finding, that instruction in digital percussion still receives in this country less attention than it deserves. My observation leads me to feel sure that the majority of medical students have not learnt to percuss properly. If this is not an error, it is a matter of very far-reaching importance. If I am right, the labour spent in studying this sign, will, if it had had no other result than that of leading to a recognition of this defect, have been labour by no means thrown away.

REFERENCES.

1 *Brit. M. J.*, 1908, ii. 298.

2 *Ibid.*, 1913, i. 1152.

3 *Lancet*, 1914, i. 161.

4 *Practitioner*, 1914.

5 *Progressive Spondylotherapy*, 1914, p. 26.

6 *Med.-Chir. Tr.*, 1904, lxxxvii. 327.

